# Research on the Spatial and Temporal Differences of China’s Provincial Carbon Emissions and Ecological Compensation Based on Land Carbon Budget Accounting

**DOI:** 10.3390/ijerph182412892

**Published:** 2021-12-07

**Authors:** Xiaodong Jing, Guiliang Tian, Minrui Li, Sohail Ahmad Javeed

**Affiliations:** 1 Business School, Hohai University, Nanjing 211100, China; jingxiaodong@hhu.edu.cn (X.J.); tianguiliang@hhu.edu.cn (G.T.); liminrui@hhu.edu.cn (M.L.); 2 School of Economics and Finance, Hohai University, Nanjing 211100, China; 3 Jiangsu Yangtze River Conservation and High-Quality Development Research Center, Nanjing 210098, China; 4 Hangzhou College of Commerce, Zhejiang Gongshang University, Hangzhou 310018, China

**Keywords:** land carbon emissions, carbon budget accounting, carbon ecological compensation, ecological compensation, temporal and spatial differences, carbon balance

## Abstract

The establishment of a complete carbon ecological compensation mechanism is of great significance for China to achieve “carbon peak and carbon neutrality” as soon as possible. From the perspective of land carbon budget accounting, this paper measures the carbon emissions and the value of carbon ecological compensation in 30 provinces in China from 2010 to 2019, by constructing a carbon ecological compensation model, and analyzes it from both time and space perspectives. The study found that: (1) during the period 2010–2019, China’s carbon absorption remained basically stable, and woodland and grassland were the main carriers of China’s land carbon absorption. The total carbon sequestration of woodland and grassland showed a pattern of being high in the west and low in the east, and the total carbon sequestration of cultivated land showed a pattern of being high in the east and low in the west. (2) Construction land is the main source of carbon emissions in China. Cultivated land carbon emissions mainly come from major agricultural provinces such as Henan and Heilongjiang, while construction land carbon emissions are mainly concentrated in energy-consuming provinces such as Shandong and Shanxi. (3) After revising the carbon compensation benchmark value, it is found that provinces such as Guangdong and Jiangsu should receive carbon ecological compensation, while provinces dominated by heavy industries such as Shanxi and Shandong need to pay corresponding carbon compensation fees. Finally, this article puts forward corresponding policy recommendations, such as that China should give full play to the role of the government and the market, accelerate the optimization and improvement of the ecological resource asset property rights system, and optimize the development and utilization of land.

## 1. Introduction

Global warming is inseparable from rising carbon dioxide concentrations. With the increasingly serious global warming problem, carbon neutrality and carbon peaking have become the inevitable choices for the sustainable development of human society and the global economy. In the process of industrialization and urbanization, the use and transformation of land by humans is an important reason for the rapid increase in carbon content in the global atmosphere, and the potential for carbon emission reduction in the process of land use is great. In the Special Report on Climate Change and Land Use, the United Nations Intergovernmental Panel on Climate Change (IPCC) discussed, in detail, the greenhouse gas flux in land use and sustainable land management, and pointed out that land plays an important role in the climate system. Only by reducing greenhouse gas emissions in all areas, including land and food, can the temperature of global warming be controlled below the target of 2 degrees Celsius. Global warming is inseparable from the increase in carbon dioxide concentration, and the carbon emissions caused by land use and land-cover changes account for 1/3 of the total carbon emissions from human activities, which greatly affects the carbon sink-carbon source of the entire region pattern.

The core issue of greenhouse gas emission reduction is the economy. The establishment of a sound carbon ecological compensation mechanism is the key to carbon emission reduction. Carbon ecological compensation has gradually become an effective means to coordinate regional ecological protection, promote economic development, and achieve carbon neutrality. In recent years, regional ecological compensation has not only become a research hotspot in the international academic community, but also an important practical issue that promotes regional fairness and coordinated green development. Ecological compensation is also called “payments for ecosystem services” [1]. It is an institutional arrangement that mobilizes the enthusiasm for ecological protection by adjusting the relationship between stakeholders. The principle of fair compensation is as follows: “Who protects, who benefits; who pollutes, who pays” [2]. After the Clean Development Mechanism (CDM), this ecological compensation is thought to be another effective emission-reduction mechanism [3], along with forest carbon compensation [4,5,6], ecological compensation for land resources [7,8,9], water compensation [10], carbon compensation technology [11], and regional carbon emission allowance allocation mechanism [12].

The current research on carbon compensation is mostly based on three perspectives: theoretical basis, ecological compensation calculation, and ecological compensation application [13]. The research on ecological compensation theory mainly includes welfare economics and public product theory, ecological capital theory and ecological justice theory. These three theories together constitute the philosophical foundation and theoretical cornerstone of ecological compensation. Welfare economics and public product theories point out that carbon emission economic activities benefit private individuals, and the resulting damages such as climate change and air pollution are borne by the entire society. This negative externality makes the prices of goods and services formed by the free market incompatible with society interest [14]. The ecological capital theory points out that ecological capital includes the physical capital value of resources and the service function and service value of the ecosystem. The ecological service value, similar to the material capital value, is scarce in social production and needs to be compensated between individuals and regions and establish a system of resource allocation [15]. The theory of ecological justice believes that environmental protection must be organically connected with social justice, and both must be paid attention to at the same time. The root of ecological justice is the injustice in the distribution of ecological resources.

In terms of ecological compensation calculation, there are currently three main methods for carbon compensation quota calculation. One is to determine the carbon compensation standard from the perspective of the value of carbon sinks. This method calculates the value generated by the increase in carbon sinks, and then uses the estimated value to determine the carbon offset standard. For example, Cupers [16] proposed the ecological value measurement method for the first time, and calculated that the total value of global ecosystem service functions is 16–54 trillion US dollars per year. Barbier [17] evaluates the value of ecosystem services from the perspective of specific ecological project operations. The second is to determine the carbon compensation standard from the perspective of compensation willingness. This method uses questionnaires to determine the carbon compensation standard [18]. For example, Chen and Jiang [19] combined the agricultural carbon measurement with the conditional value evaluation method and used the farmer’s data obtained from the survey to determine the amount of ecological compensation for the farmer’s low-carbon agricultural production. The third is to determine the carbon compensation standard from the perspective of carbon balance, by measuring the carbon sink or net carbon sink in production activities and measuring the carbon ecological compensation standard by the profit and loss of the carbon sink. If the carbon sink is surplus, then the area should receive an ecological compensation amount, otherwise the compensation amount should be paid [20,21,22,23,24,25].

The three carbon offset calculation methods mentioned above all have certain advantages and disadvantages. From the perspective of determining the carbon compensation standard from the value of carbon sinks, the advantage is that the carbon compensation quota can be measured only by considering the increase in carbon sinks, but the disadvantage is that the generation of carbon emissions is ignored. From the perspective of compensation willingness, the advantage of this standard is that it can reflect the true psychological price of the carbon sink transferor and the transferee to a certain extent, but the disadvantage is that it is easily affected by the trading parties’ education, experience, family situation, gender, age, etc. Carbon compensation quota calculation from the perspective of carbon balance can not only fully consider carbon emissions and carbon sinks, but also ensure that the calculation of carbon emissions and carbon sinks is objective and reliable, and will not be affected by external factors; this is conducive to the development of unified carbon compensation in different provinces.

In the application field of ecological compensation, scholars focused on engineering construction compensation [26], forest carbon compensation [27,28], agricultural carbon compensation [29], forestry carbon compensation [30], carbon sink fishery compensation [31], tourism carbon compensation [32], reservoir development carbon compensation [33], carbon sink value evaluation [34,35], regional carbon ecological compensation [36] and carried out a significant amount of research, which provides a useful reference for the construction of carbon compensation theories and methods. But overall, regional carbon compensation is still in the exploratory research stage, and the research on carbon compensation standards and models across provinces needs to be further strengthened.

China is a country with a vast territory. At the same time, the contradiction between China’s regional economic development and ecological environmental protection is also prominent. The question of how to introduce the carbon ecological compensation mechanism into regional economic development and not only ensure high-quality economic and social development, but also promote equitable development between provinces, has become a social problem that needs to be solved urgently. From the provincial scale, the economic level, industrial structure, climate regulation, ecological resource endowment, and other spatial differences of various provinces are obvious. Therefore, it is of great practical significance to study the spatial-temporal differences in carbon emissions and ecological compensation based on land carbon budget accounting. The innovative aspect of this article is its focus on land production activities, measure the carbon emissions of China’s provinces at the national level, use the pearl growth curve method to correct the carbon absorption, and analyze the carbon emissions between provinces from the perspective of carbon source and carbon sink, which excludes the influence of human factors on the experimental results, and makes the results fairer. Secondly, this article takes the differences in economic development of various provinces into consideration to ensure the feasibility of regional ecological justice and carbon ecological compensation to the greatest extent. The potential contributions of this paper are as follows: This paper takes 30 provinces of China from 2010 to 2019 as research objects and links the amount of ecological compensation with the market price of carbon emission rights trading, and the research conclusions can provide a relevant theoretical basis for ecological compensation and the initial allocation of carbon emission rights between provinces. At the same time, the “China Plan” can also provide some experience for other similar countries.

The study is organized as follows: The second section shows the paper’s model and data. The third section shows the model’s calculation results. The fourth section is the experimental discussion. The fifth section includes research conclusions and relevant policy recommendations.

## 2. Materials and Methods

**Figure 1:**
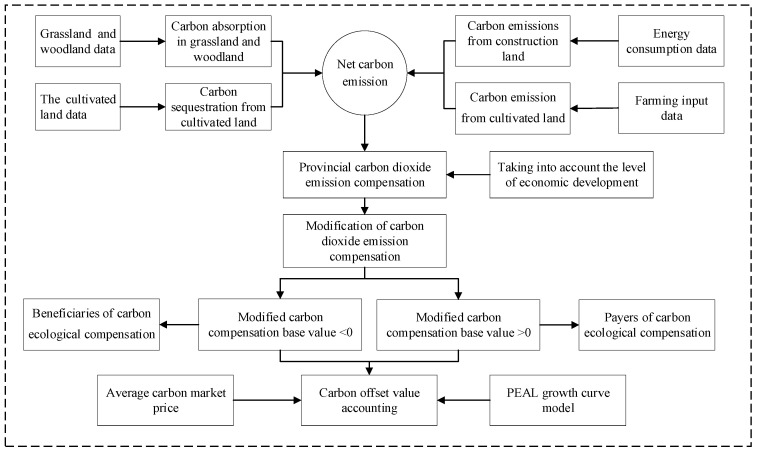
Framework of ecological compensation for carbon emissions in China.

This paper takes 30 provinces in China as the research object, and conducts carbon emission and carbon absorption research on four main land types: forest land, grassland, cultivated land and construction land. The amount of carbon absorption mainly comes from land absorption such as forest land, grassland and arable land. Carbon emissions mainly include carbon emissions from construction land and carbon emissions from cultivated land. Among them, arable land is both a carbon source and a carbon sink. Due to missing data, the scope of the study does not include Hong Kong, Macau, Taiwan and Tibet Autonomous Region. The research framework of ecological compensation for carbon emissions in China is shown in Figure 1.

### 2.1. Methodology

#### 2.1.1. Carbon Absorption Calculation Method

(1) The calculation method for carbon absorption of woodland and grassland through photosynthesis is as follows:

(1) $$C_{m}=S_{m}\times a_{m}$$

In the formula (1), $C_{m}$ is the carbon absorption of m land types, $S_{m}$ is the area of m land types, and $a_{m}$ is the carbon absorption coefficient of m land types. The carbon absorption coefficients of woodland and grassland are 3.81 t/hm^2^ and 0.91 t/hm^2^, respectively [37].

(2) For the carbon absorption of arable land, the following formula is used to calculate [24]:

(2) $$CI_{crop}=\sum_{i}CI_{crop-i}\times \left(1-P_{water-i}\right)\times \frac{Y_{eco-i}}{H_{crop-i}}$$

where $CI_{crop}$ is the photosynthetic carbon absorption during the growth period of the crop, $CI_{crop-i}$ is the carbon absorption of the *i* crop, $C_{crop-i}$ is the carbon absorption of the *i* crop through photosynthesis unit organic matter (dry weight), $P_{water-i}$ is the water content of the *i* crop, $Y_{eco-i}$ is the economic output of the *i* crop, $H_{crop-i}$ is the economic coefficient of the *i* crop. This paper mainly calculates the carbon absorption of crops such as rice, wheat, corn, beans, potatoes, hemp, sugar beet and flue-cured tobacco. At present, the economic coefficient and carbon absorption rate of crops commonly used in academic circles generally come from the research results of Wang and Li [37,38] and the average moisture content comes from the results of Fang et al., [39]. The economic coefficient, moisture content and carbon absorption rate of China’s main crops are shown in the Table 1.

#### 2.1.2. Carbon Emission Calculation Method

(1) Cultivated land carbon emissions.

Cultivated land acts as a carbon source for human crop farming activities. Cultivated land carbon emissions are mainly derived from the following five aspects: one is the direct or indirect carbon emissions caused by the use of chemical fertilizers; the second is the carbon emissions caused by the use of pesticides; the third is the production of crop planting areas and the use of agricultural machinery; the fourth is the carbon emissions caused by the agricultural irrigation process; and the fifth is the carbon emissions caused by the use of agricultural film. The formula for calculating carbon emissions from cultivated land is as follows:

(3) $$E_{t}=GM+TN+(SO+PQ)+FR+AU$$

In the formula (3), Et is arable land carbon emissions, G, T, S, P, F, A are, respectively, the amount of chemical fertilizers, pesticides, crop planting area, total power of agricultural machinery, irrigation area and agricultural film usage, M, N, O, Q, R, and U are the corresponding conversion coefficients. The values of M and N are from the Oak Ridge National Laboratory (ORNL) [40], which are 0.8956 kg/kg and 4.9341 kg/kg, respectively. O, Q, and R are 16.47 kg/hm^2^, 0.18 kg/kW, 266.48 kg/hm^2^, respectively [41]. U is 5.18 kg/hm^2^, and the data comes from the Institute of Agricultural Resources and Ecological Environment (IREEA), Nanjing Agricultural University.

(2) Carbon emissions from construction land.

There are currently three main methods for calculating carbon dioxide emissions, namely the input-output method [42], the life cycle method [43] and the carbon emission coefficient method [44]. Since the input-output method only releases data every 5 years, the data cannot be coherent in research. The life cycle method is to track the environmental impact of construction land during the entire life cycle. It has higher requirements for data, and its calculation process is complicated, which increases the uncertainty of the results. Therefore, this article uses the reference method in the 2006 IPCC National Greenhouse Gas Inventory Guidelines to calculate carbon emissions from construction land, and selects eight types of coal, coke, crude oil, fuel oil, gasoline, kerosene, diesel and natural gas as the main energy sources for calculation. The specific calculation formula for total carbon dioxide emissions is as follows:

(4) $$CO_{2}=\sum_{i=1}^{8}CO_{2,i}=\sum_{i=1}^{8}E_{i}\times NCV_{i}\times CEF_{i}\times COF_{i}\times \frac{44}{12}$$

In the formula (4), *i* is the *i* type of fossil fuel, which mainly selects eight kinds of fossil energy, such as coal, coke, crude oil, gasoline, kerosene, diesel, fuel oil, and natural gas as counted in the “China Energy Statistical Yearbook”. $E_{i}$, $NCV_{i}$, $CEF_{i}$ and $COF_{i}$ are, respectively, the consumption, low calorific value, carbon content and carbon oxidation coefficient of the *i* kind of fossil fuel. 44 and 12 are the chemical molecular weights of carbon dioxide and carbon, respectively.

#### 2.1.3. Calculation of Carbon Compensation Value of Land Use

Net carbon emissions (the difference between carbon sources and carbon sinks) are used as the basis for confirming the baseline value of carbon compensation. If the carbon sink value of a province is higher than the carbon source value, it can reflect that the province’s ecological carbon sequestration capacity is better. This province can absorb carbon emissions not only from its own province, but also from nearby provinces, and should be compensated by funds. On the contrary, when the carbon source value is higher than the carbon sink value, the province should spend funds to compensate other provinces. The specific calculation formula of carbon compensation is as follows:

(5) $$L_{f}=E_{cf}-S_{cf}$$

In the formula (5), $L_{f}$ is the net carbon emission of *f* province; $E_{cf}$ is the carbon emission of *f* province, and $S_{cf}$ is the carbon absorption of *f* province. When $L_{f}>0$, the province should pay carbon compensation funds to other provinces, and when $L_{f}<0$, the province should receive carbon compensation funds from other provinces.

In reality, the economic development status and development model of different provinces are different from each other, and the net carbon emissions are quite different. If only the net carbon emissions are used to calculate the carbon compensation value, the calculation results will be somewhat biased. In order to bring the carbon compensation results closer to the real situation, this study sets a carbon emission threshold for each province, the specific formula is as follows:

(6) $$P_{f}=ECC\times D=\frac{G_{f}}{G}/\frac{C_{f}}{C}\times D$$

In the formula (6), *ECC* is the economic contribution coefficient of carbon emissions, *D* is the national average carbon emissions from 2010 to 2019, $G_{f}$ and *G* are provincial *GDP* and national *GDP*, respectively, and $C_{f}$ and *C* are the carbon emissions of *f* Province and China, respectively.

In addition to the obvious differences in net carbon emissions, there are also temporal and spatial differences in carbon emission intensity between different provinces. This study combines the carbon emission intensities of different provinces in 2010 and 2019 to revise carbon emissions as follows:

(7) $$E_{cf}^{1}=E_{cf}\times \left(\frac{G_{t1-f}}{G_{t2-f}}-\frac{G_{T1}}{G_{T2}}+1\right)\times \frac{G_{t1-f}}{G_{T}}$$

where $E_{cf}^{1}$ is the revised carbon emission of *f* province, $G_{t1-f}$ and $G_{t2-f}$ are the carbon emission intensity of *f* province in 2019 and 2010, $G_{T1}$ and $G_{T2}$ are the total carbon emission intensity of China in 2019 and 2010, and $G_{T}$ is the average carbon emission intensity of all provinces across the country in 2019. The revised carbon compensation benchmark values are as follows:

(8) $$L_{f}^{1}=E_{cf}^{1}-S_{cf}-P_{f}$$

In the formula (8), $L_{f}^{1}$ is the revised carbon compensation benchmark value. The advantage of the modified model is that the carbon compensation rate and carbon emission intensity are closer to the actual situation. If $L_{f}^{1}<0$, the province should obtain carbon compensation funds, and if $L_{f}^{1}>0$, then this province should pay carbon offset funds to others.

#### 2.1.4. Carbon Offset Value Calculation Method

On the basis of Yu’s research [23], the model is appropriately revised, and the calculation method of the carbon compensation value is obtained as follows:

(9) $$M_{f}=\left|L_{f}^{1}\right|\times \partial \times \gamma =\left|E_{cf}^{1}-S_{cf}-P_{f}\right|\times \partial \times \gamma$$

In the formula (9), $M_{f}$ is the carbon compensation fund that should be obtained or paid by the region (yuan); ∂ is the unit carbon price (yuan/ton); γ is the carbon compensation coefficient.

Among them, the carbon price ∂ is based on the national average price of carbon emissions trading from 2013 to 2019 (China’s carbon emissions trading began to pilot in 2013), and the data comes from the daily data of China’s carbon emissions trading. Carbon price calculation formula is as follows:

(10) $$\partial =(\sum M_{f}/\sum V_{f})\times GP_{f}/GP$$

In the formula (10), $\sum M_{f}$ is the total amount of national carbon emission rights transactions from 2013 to 2019 (yuan). $\sum V_{f}$ is the total amount of national carbon emission rights transactions from 2013 to 2019 (tons). $GP_{f}$ is the per capita GDP of each province in 2019 (yuan/person) and GP is the national GDP per capita in 2019 (yuan/person).

Different provinces have different levels of economic development, which will lead to differences in each province carbon offset capabilities. Therefore, in order to further consider the actual payment capacity of each region, it is necessary to determine the carbon compensation coefficient according to the economic development level of different regions. This paper uses an improved Pearl growth curve model (S-shaped growth curve) to express:

(11) $$\gamma_{f}=A_{f}/\left(1+ae^{-bt}\right)$$

In the formula, $\gamma_{f}$ represents the carbon compensation coefficient of the *f* province, $A_{f}$ represents the carbon compensation capacity of the *f* province, namely, the *f* provinces GDP share of the country’s GDP. *a* and *b* are constants, here we take 1. *t* is the Engel coefficient of China in that year.

### 2.2. Variable Description and Data Sources

The data sample of this article takes 30 provinces in China as the research object. Due to lack of data, the research scope does not include Hong Kong, Macau, Taiwan and Tibet Autonomous Region. The time span of the research object in this article is 2010–2019. In order to exclude the impact of price factors on the sample, all variables involving prices are converted at constant prices in 2010. Data on construction land energy consumption, arable land carbon emissions, and arable land carbon absorption are from the “China Environmental Statistical Yearbook”, “China Energy Statistical Yearbook” (CESC) [45], and “China Statistical Yearbook” [46]. GDP and Engel’s coefficient are from the National Bureau of Statistics [46]. The carbon emissions coefficient was collected from the IPCC carbon emissions calculation guide (IPCC, 2006). The price of carbon emissions trading comes from the carbon emissions trading network [47].

## 3. Results

### 3.1. Temporal and Spatial Differences in Carbon Absorption

#### 3.1.1. Temporal and Spatial Differences in Carbon Absorption of Forest Land and Grassland

**Figure 2:**
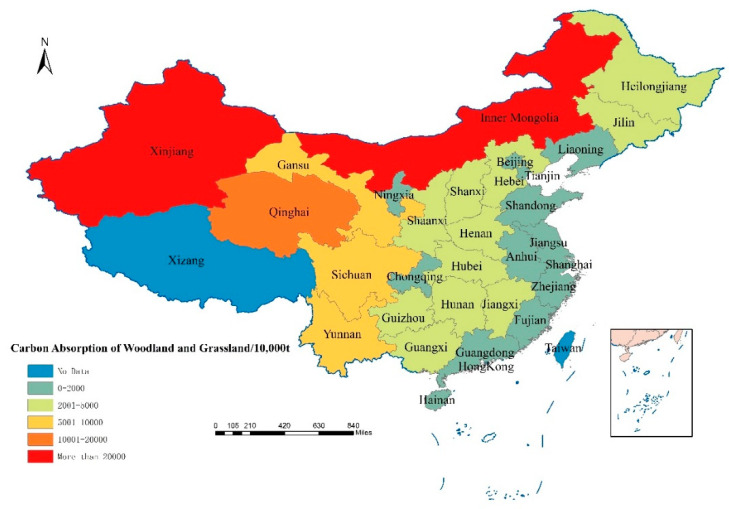
The total carbon absorption of China’s woodland and grassland in 2019.

According to the total amount of carbon absorption of woodland and grassland in 30 provinces of China, it is found that the total area of grassland and forest land in China remained basically unchanged in recent years, and the overall carbon absorption of woodland and grassland remained stable. In 2019, China’s grassland and woodland carbon absorption totaled 1854.17 million tons (Figure 2), of which Inner Mongolia and Xinjiang provinces had the largest carbon absorption, with 371.96 million tons and 270.26 million tons, respectively, accounting for 36.64% of the country’s total grassland and woodland carbon absorption. Tianjin, Beijing and Shanghai have the least carbon absorption from woodland and grassland, with 0.69 million tons, 1.86 million tons and 0.34 million tons, respectively, accounting for only 0.16% of the national total carbon absorption from woodland and grassland. In addition, China’s woodland and grassland carbon uptake is geographically high in the west and low in the east. The total carbon uptake of forestland and grassland in Qinghai is 171.67 million tons, followed by Sichuan, Gansu, and Yunnan, with 96.19 million tons, 84.51 million tons and 72.26 million tons, respectively. The carbon absorption of forest land and grassland in the eastern coastal provinces is relatively small. For example, the amount of carbon absorbed by forest land and grassland in Jiangsu was 1.95 million tons, and that in Shandong was 7.73 million tons.

During the period 2010–2019, China’s total carbon absorption of forest land was 14.94 billion tons, which contributed 68.55% to the total carbon sequestration; the total carbon absorption of grassland was 3.57 billion tons, which contributed 16.37% to the total carbon sequestration. The total contribution rate of woodland and grassland carbon absorption to the total carbon sequestration is 84.92%. Therefore, woodland and grassland are the main carriers of land-use carbon absorption in China.

#### 3.1.2. Temporal and Spatial Differences in Carbon Absorption of Cultivated Land

**Figure A1:**
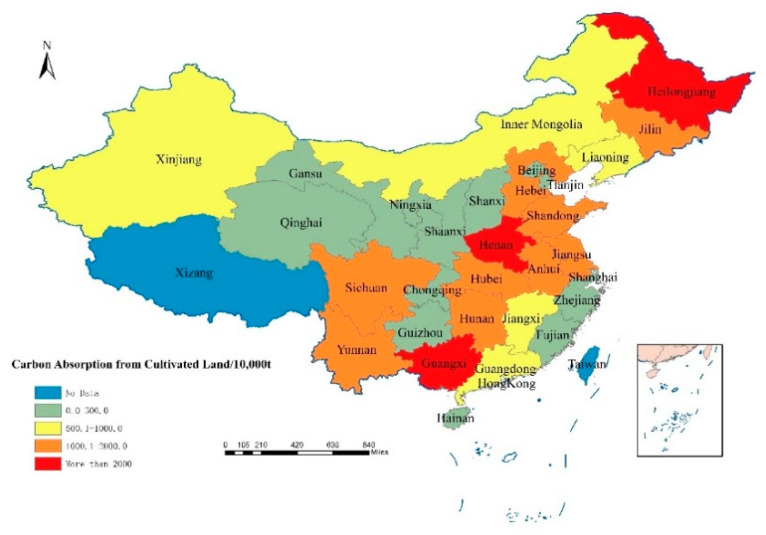
The total carbon absorption of arable land in China’s provinces in 2010.

**Figure A2:**
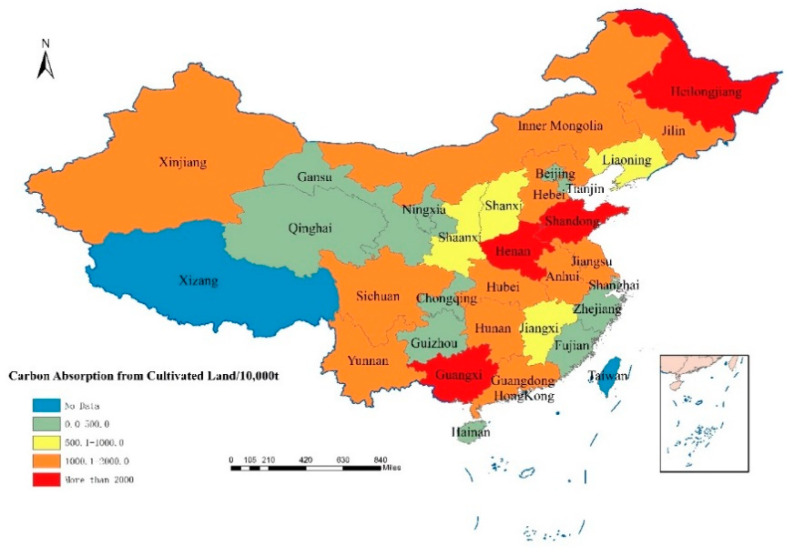
The total carbon absorption of arable land in China’s provinces in 2019.

Through the calculation of the carbon uptake results of the cultivated land of 30 provinces in China from 2010 to 2019, the results are tabulated and shown in Table 2. Due to space limitations, the results of 2010, 2015 and 2019 are listed in the article for display. Overall, China’s total carbon sequestration of arable land has increased year by year in recent years (Table 2). In 2010, China’s total arable land carbon absorption was 271.69 million tons, and in 2019 it was 320.59 million tons, with an average annual growth rate of 1.67%. In 2010 (Figure A1), the top five provinces in China’s arable land carbon absorption were Guangxi (30.67 million tons), Henan (25.03 million tons), Heilongjiang (20.22 million tons), Shandong (19.66 million tons) and Sichuan (13.82 million tons). The reason why the carbon uptake of Guangxi’s cultivated land is the highest in China is mainly due to the high yield of local fruits and sugar crops. Henan is the main production area of wheat and corn in China. In 2010, Henan produced 30.82 million tons of wheat and 16.35 million tons of corn. In 2015, the total carbon sequestration of cultivated land in Inner Mongolia rose to 12.66 million tons, an increase of 35.05% compared to 2010. The total carbon absorption of cultivated land in Guangdong exceeded 10 million tons, reaching 10.64 million tons. The total carbon absorption of cultivated land in Shanxi, Shaanxi, Chongqing and other provinces remained basically unchanged. In 2019 (Figure A2), Inner Mongolia ranked fifth in the total carbon absorption of China’s arable land, with a total of 17.11 million tons, an increase of 82.53% from the 9.37 million tons in 2010, with an average annual growth rate of 6.20%, which greatly exceeded the national average growth level. Mt means 10,000 tons.

### 3.2. Temporal and Spatial Differences in Carbon Emissions

#### 3.2.1. Temporal and Spatial Differences in Carbon Emissions from Cultivated Land

**Figure A3:**
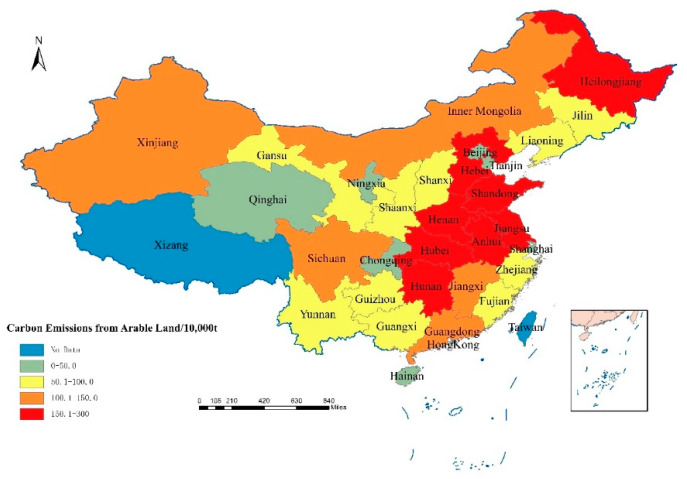
Total carbon emissions from cultivated land in China’s provinces in 2010.

**Figure A4:**
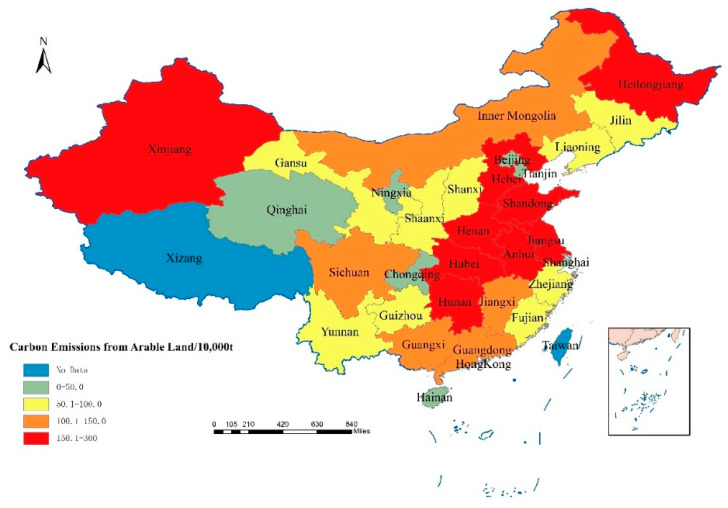
Total carbon emissions from cultivated land in China’s provinces in 2019.

By calculating the total carbon emissions of cultivated land in China from 2010 to 2019, it is found that in the past 10 years, the total carbon emissions of China’s cultivated land remained stable as a whole (Table 3). In 2010, the total carbon emissions from cultivated land nationwide was 31.22 million tons (Figure A3), and in 2019 it was 31.73 million tons (Figure A4). In terms of provinces, the top five provinces with carbon emissions from cultivated land in 2010 were Shandong (2.46 million tons), Henan (2.35 million tons), Hebei (1.92 million tons), Anhui (1.80 million tons) and Heilongjiang (1.73 million tons). Cultivated land in Ningxia, Tianjin, Shanghai, Qinghai, and Beijing has relatively small carbon emissions. In 2015, four provinces including Henan, Shandong, Heilongjiang, and Anhui accounted for more than 2 million tons of carbon emissions from arable land. Xinjiang’s carbon emissions from cultivated land in 2015 were 1.67 million tons, an increase of 29.67% from 1.73 million tons in 2010. In 2015, carbon emissions from cultivated land in Yunnan exceeded 1 million tons for the first time. In 2019, Heilongjiang’s arable land carbon emissions reached 2.34 million tons, an increase of 35.56% compared to 2010, which was 1.73 million tons, with an average annual growth rate of 3.09%. Beijing, Tianjin and Shanghai have basically not lost their agricultural functions, and the carbon emissions of arable land declined year by year.

#### 3.2.2. Temporal and Spatial Differences in Carbon Emissions from Construction Land

**Figure A5:**
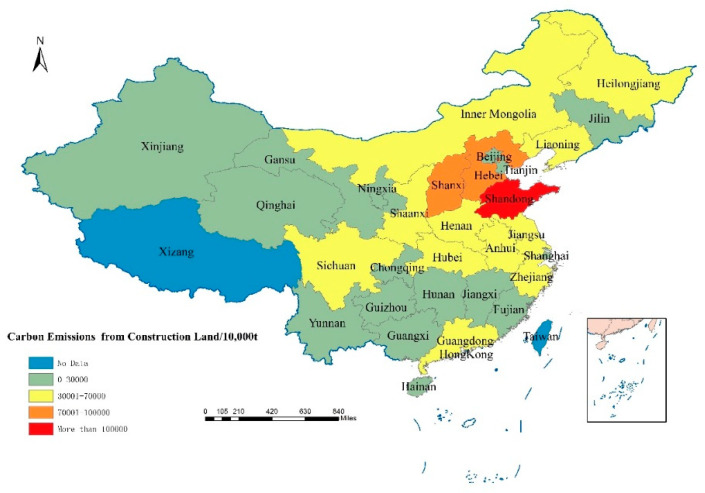
Carbon emissions from construction land in China’s provinces in 2010.

**Figure A6:**
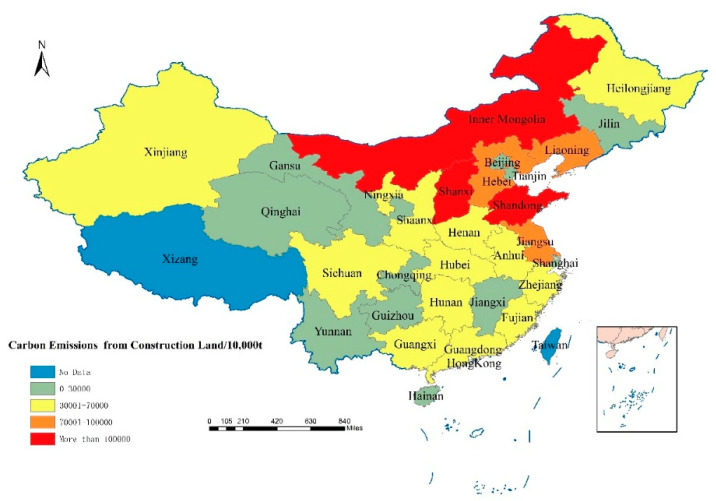
Carbon emissions from construction land in China’s provinces in 2019.

The transformation of construction land by mankind mainly comes from the burning of fossil fuels. Through the measurement of carbon emissions generated by the consumption of eight major fossil energy sources, it can be seen that during the study period, China’s carbon emissions from construction land showed a steady growth stage. In 2010, China’s total carbon emissions from construction land was 10.92 billion tons (Figure A5), and in 2019 it was 13.81 billion tons (Figure A6), with an average annual growth rate of 2.38%. From a geographical perspective, China’s energy consumption as a whole shows a trend of “high in the east and low in the west, high in the north and low in the south”. This distribution characteristic is generally consistent with the resource endowment structure and economic development level of China’s provinces. Bt means billion tons.

From the analysis of various provinces, the carbon emissions of construction land in various provinces have obvious differences, and they are gradually extending in the energy-rich areas in the north, forming high energy consumption regions represented by Shaanxi, Shanxi, Shandong, and Inner Mongolia (Table 4). In 2015, the carbon emissions of construction land in Shandong, Hebei, Shanxi, Jiangsu, and Inner Mongolia were 1.33 billion tons, 882.59 million tons, 835.79 million tons, 831.56 million tons and 783.58 million tons, respectively, (Table 4). These provinces’ carbon emissions of construction land accounted for 37.42% of China’s total building carbon emissions. In 2019, there were 3 provinces in China with construction land carbon emissions exceeding 1 billion tons, including Shandong (1.489 billion tons), Shanxi (1.133 billion tons), and Inner Mongolia (1.046 billion tons). At the same time, there are 3 provinces with construction land carbon emissions exceeding 800 million tons, including Hebei (933.10 million tons), Jiangsu (835.88 million tons) and Liaoning (814.87 million tons).

### 3.3. Accounting Analysis of China’s Provincial Carbon Budget

By measuring the carbon absorption of forest land, grassland and arable land and the carbon emissions of arable land and construction land in 30 provinces in China, it can be concluded that China’s total carbon emissions are significantly greater than carbon absorption, and it has not yet reached the ideal state of carbon neutrality. From the previous analysis, it can be seen that many underdeveloped regions in China have lost many economic development opportunities for the overall ecological environment of the country, and, at the same time, the ecological service value provided by them is shared by other parts of the country. This regional division of economic benefits and the global ecological service value sharing will result in a lack of incentives for environmental protection in underdeveloped areas. Therefore, in order to continue to promote the construction of national ecological civilization and alleviate the imbalance of the ecological mechanism caused by the uneven and inadequate regional economic development, it is extremely necessary to compensate for the cost of lost opportunities in the underdeveloped regions and other loss due to environmental protection.

By incorporating the economic development status and ecological resource conditions of various provinces into the carbon ecological compensation factor, the revised carbon compensation benchmark value can be fairer and more operable. The revised carbon compensation benchmark values are shown in Table 5. When the carbon compensation benchmark value is greater than 0, it means that the province needs to pay carbon ecological compensation fees. If the carbon compensation benchmark value is less than 0, it means that the province should receive carbon ecological compensation funds.

**Figure 3:**
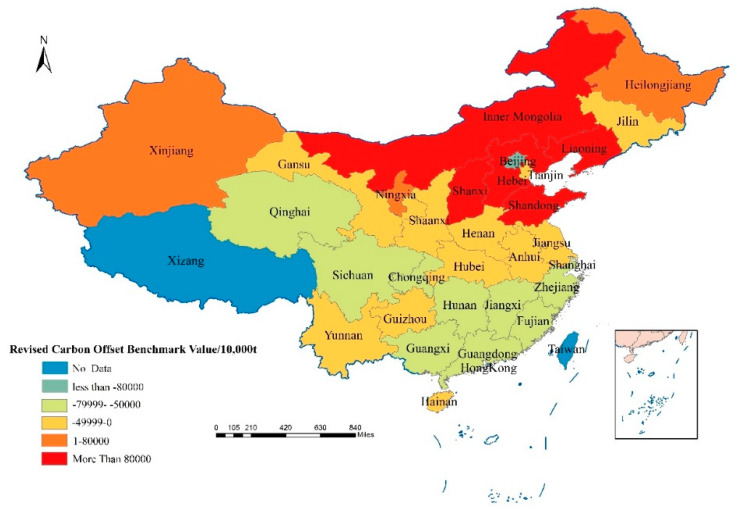
Revised carbon offset benchmark values of all provinces in China in 2010.

**Figure 4:**
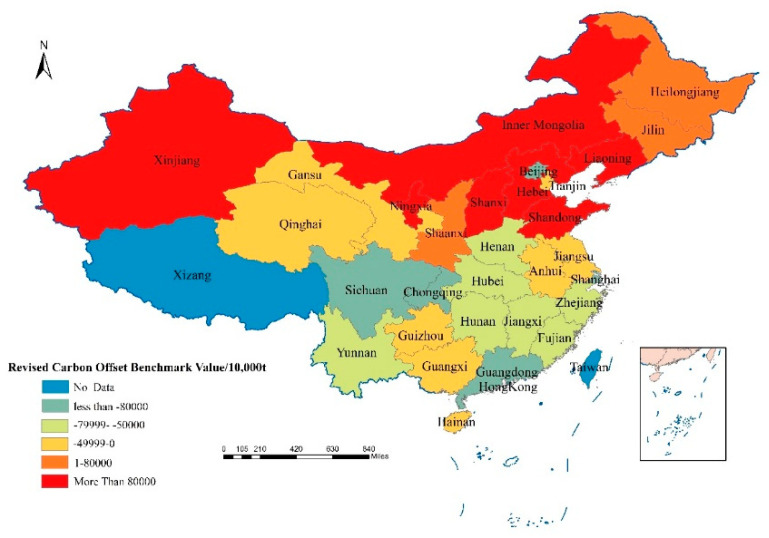
Revised carbon offset benchmark values of all provinces in China in 2019.

It can be seen from Table 5 that the underdeveloped regions represented by Sichuan, Yunnan, Hunan, Henan, Hubei, and Guangxi have higher net carbon absorption, while Inner Mongolia, Shanxi, Shaanxi, and Shandong have higher net carbon emissions (Table 5). Generally speaking, provinces with large net carbon absorption are mostly large agricultural provinces or economically underdeveloped provinces (Figure 3 and Figure 4). This type of province has large areas of arable land or high-grade vegetation coverage. The provinces with large net carbon emissions are generally concentrated in China’s northern energy-rich provinces, such as Inner Mongolia, Shanxi, and Shaanxi, which are all provinces with large coal reserves. Over-exploitation and consumption of energy make these provinces have large carbon emissions and have a negative impact on the environment.

### 3.4. Accounting Analysis of the Value of Carbon Offsets in China’s Provinces

By combining the price of China’s carbon emissions trading market and incorporating regional economic development differences into the scope of the model, the calculated results of the spatial carbon offset value of China’s provinces are shown in Table 6. The results found that there are large spatial differences in the value of carbon offsets between provinces in China.

**Figure 5:**
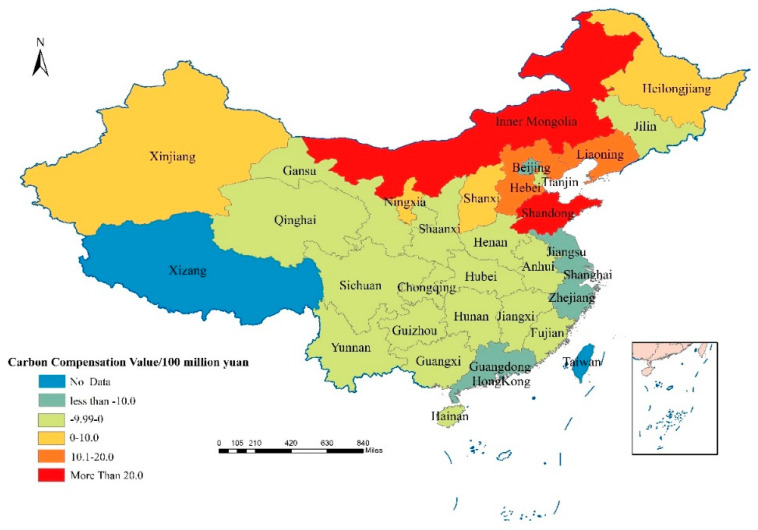
The calculation results of the carbon offset value of China’s provinces in 2010.

**Figure 6:**
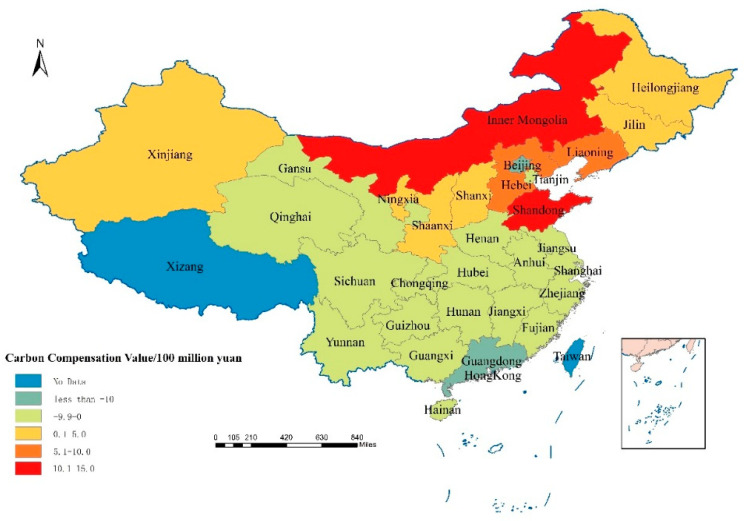
The calculation results of the carbon offset value of China’s provinces in 2019.

In 2010, Qinghai, Gansu, Sichuan, Chongqing, Yunnan, Guizhou located in the western region, and Jilin, Beijing, Tianjin, Jiangsu, Shanghai, Zhejiang, Fujian, Guangdong and Guangxi located in the eastern region should receive higher carbon ecological compensation (Figure 5). The province that should receive the highest carbon ecological compensation is Guangdong with 3.33 billion yuan, followed by Jiangsu. In 2010, Jiangsu should receive 1.71 billion yuan for carbon ecological compensation (Table 6). The provinces that need to pay carbon ecological compensation in 2010 are Inner Mongolia, Hebei, Shanxi, Liaoning, Heilongjiang, Shandong and Ningxia (Figure 5). Among them, Shandong has the highest amount of ecological compensation payable, with a carbon ecological compensation amount of 3.33 billion yuan, followed by Inner Mongolia, Liaoning Province and Hebei, with payable amounts of 2.72 billion yuan, 1.99 billion yuan and 1.64 billion yuan, respectively, (Table 6). In 2019, a total of 20 provinces in China should receive carbon ecological compensation, and 10 provinces need to pay carbon ecological compensation fees (Figure 6). Among them, the province with the highest amount of carbon ecological compensation is Guangdong, which is 1.38 billion yuan, and the province with the highest amount of carbon compensation that should be paid is Shandong, with a total amount of 1.50 billion yuan (Table 6). It should be noted that although some provinces have relatively large carbon emissions, their economic contribution coefficients are relatively high, such as Beijing, Tianjin, and Shanghai. Therefore, after the economic contribution coefficient of carbon emissions is revised, the actual allowances for their emissions are allowed to be higher. As a result, the amount of carbon ecological compensation that should be obtained will increase or the amount of carbon emissions that should be paid will decrease. Conversely, some provinces with low economic contribution coefficients will receive fewer carbon emission allowances after the carbon emission benchmark value is revised, so the amount of carbon ecological compensation they should receive will be reduced, or the fees that they should be paid will be increased.

This paper links the price of carbon emissions with the capital market, and fully considers the actual differences in the economic development level of each province in the calculation of the carbon ecological compensation value, which can ensure the relative fairness of the carbon ecological compensation schemes in each province to a greater extent, thereby promoting inter-provincial obtain higher quality development.

## 4. Discussion

(1) Based on the perspective of land carbon balance, this paper uses ArcGIS to visually analyze the land carbon emissions, carbon absorption and ecological compensation in 30 provinces in China. The results of this paper are consistent with Zhong’s research results [48] and Miao’s research results [49]. The research results of Miao [49] showed that the provinces with higher net carbon emissions in China in 2015 were Shandong, Hebei, Jiangsu, Shanxi and Liaoning, which are the same as our results. However, the improvement of this study compared to Yang’s research is that the weighted average price of carbon emission prices is used instead of the average of the maximum and minimum carbon emission prices during the study period in accordance with Miao [49]. On the one hand, using the maximum and minimum values to measure the value of carbon ecological compensation will have a large deviation error. On the other hand, adopting the carbon emission price without considering the carbon emission transaction volume can easily cause the distortion of the carbon price, which is inconsistent with the actual situation.

(2) In recent years, China’s total land carbon absorption and carbon emissions have shown an upward trend year by year, of which the total land carbon absorption growth rate is relatively slow, and the land carbon emission growth rate is relatively fast. The carbon absorption of land mainly comes from the carbon absorption of forest land and grassland, and the carbon emission mainly comes from the carbon emission of construction land. In 2010, China’s total land carbon absorption was 1.73 billion tons, and in 2019 it was 1.79 billion tons. The average annual growth rate of total carbon absorption was 0.39%. In 2010, China’s total land carbon emissions were 11.00 billion tons, and in 2019, total carbon emissions were 13.85 billion tons, with an average annual growth rate of 2.64%. The rapid growth of land carbon emissions indicates a significant increase in total energy consumption in the process of rapid urbanization and industrialization. At the same time, because the growth rate of land carbon emissions is greater than the growth rate of land carbon absorption, China’s pressure to reduce carbon emissions is also increasing. It should be noted that the contribution of land carbon absorption to the reduction of net carbon emissions is not high. Therefore, it is still necessary to focus on the adjustment of industrial structure and the transformation of energy consumption structure to achieve the decoupling of GDP and carbon emissions as soon as possible.

(3) At present, ecological compensation research mainly focuses on theoretical policies, compensation methods, and compensation necessity, but there are relatively few studies on the standard system and little quantitative research on overall regional ecological compensation judgments. In addition, the determination of ecological compensation standards in the world has not yet formed a set of universal ecological compensation theoretical basis and ecological compensation standards. In the process of cross-regional ecological compensation, the government lacks a suitable indicator system for objective evaluation, and the transaction cost is relatively high.

(4) Eco-compensation research based on land carbon balance will provide a certain empirical reference for China’s eco-compensation practice. Ecological compensation based on the balance of land carbon budget suggests the use of carbon emission indicators as a scarce resource and carbon absorption capacity as a means of income. Taking advantage of the differences in carbon emissions and carbon absorption among Chinese provinces, through negotiation and market transactions, a reasonable transaction price can be formed, so that ecological services will move from free to paid. At present, carbon trading and compensation are still in a period of exploration in China, but for the global carbon market, China will soon participate in the world carbon trading market. Therefore, to develop the carbon market as soon as possible and actively explore experience is conducive to the establishment of China’s carbon market and China’s active position in the world’s carbon market.

(5) Since 2013, China’s carbon emissions trading has been piloted in 8 provinces, mainly covering energy-intensive industries such as power generation, building materials, and steel. However, China’s carbon emissions trading has been ineffective in the pilot phase of the market. From August 5, 2013 (the earliest date of carbon emissions trading available in China) to December 31, 2019, a total of 187.68 million tons of CO_2_ were traded in China’s carbon emissions trading, with an average trading volume of 22.05 thousand tons. The market for carbon emission rights is seriously lacking in liquidity. At the same time, the carbon trading volume of different pilot provinces is quite different. For example, Hubei has traded a total of 60.07 million tons of carbon dioxide in the past 7 years, accounting for 32.01% of the total carbon emissions trading market in China, while the carbon emission rights trading in Beijing, Fujian, Tianjin and Chongqing only accounted for 7.08%, 4.24%, 1.62%, and 4.50% of China’s carbon emission rights trading market, respectively. Secondly, the trading prices of carbon emission rights in different provinces are quite different, and the fluctuation range is also different. Specifically, the carbon price in Beijing is relatively high. During the study period, the average carbon price in Beijing was 59.82 yuan/ton, and the price was relatively stable. But the carbon price in Chongqing is only 5.68 yuan/ton, which is the lowest price compared to other provinces. Additionally, the carbon price in Shanghai showed a downward trend and then an upward trend. Its highest price was 143.99 yuan/ton and its lowest price was 4.00 yuan/ton. Obviously, large fluctuations in carbon prices are extremely unfavorable for the determination of the value of carbon ecological compensation. Although this paper uses the average transaction price of carbon emission rights in the past 7 years to determine the carbon price, the development of China’s carbon market is still insufficient, and carbon prices are distorted to a certain extent. If China’s carbon emissions trading market can be further improved in the later period, the determination of the carbon offset amount can be closer to reality.

## 5. Conclusions

Based on the perspective of land carbon accounting, this paper uses the carbon emission coefficient method to calculate the carbon emissions and carbon absorption of 30 provinces in China from 2010 to 2019, and uses the Pearl growth curve method to revise the carbon emissions of each province. Based on the carbon balance of each province, this article analyzes the differences in carbon emissions of each province from two dimensions of time and space through ecological compensation coefficients and economic contribution coefficients, and conducts carbon ecological compensation research on 30 provinces with net carbon emissions as a benchmark value. According to the research results, the following conclusions are obtained:

(1) From the perspective of carbon absorption, forest land and grassland are the main carriers of China’s land carbon absorption, and their contribution to carbon absorption reaches 84.92%. During the period 2010–2019, China’s carbon absorption remained stable as a whole. In 2010, the total amount of carbon sequestered nationwide was 1.72 billion tons, and in 2019 it was 1.79 billion tons, with an average annual growth rate of 0.38%. In terms of geographical distribution, the total carbon sequestration of woodland and grassland presents a pattern of high in the west and low in the east, and the total carbon sequestration of arable land presents a pattern of high in the east and low in the west. The woodland and grassland in Qinghai, Sichuan, Gansu and other provinces have higher carbon sequestration, while the woodland and grassland in Jiangsu and Shandong provinces have less carbon sequestration. Guangxi, Henan, Heilongjiang, Shandong and other provinces have higher carbon sequestration in cultivated land, while Gansu, Qinghai, Ningxia and other provinces have lower carbon sequestration in cultivated land.

(2) From the perspective of carbon emissions, carbon emissions from construction land are the main source of carbon emissions in China, and the proportion of carbon emissions from cultivated land is relatively low. In 2010, China’s total carbon emissions were 10.95 billion tons and 13.85 billion tons in 2019, with an average annual growth rate of 2.37%. From the perspective of spatial distribution, China’s carbon emissions also show obvious geographic differences. Carbon emissions from cultivated land are mainly concentrated in major agricultural provinces such as Heilongjiang, Henan, and Shandong, and carbon emissions from construction land are mainly concentrated in energy-consuming provinces, such as Shandong, Inner Mongolia, Shanxi and Shaanxi.

(3) From the perspective of net carbon emissions, China’s net carbon emissions have gradually increased in recent years. In 2010, the national net carbon emissions were 9.23 billion tons and 12.06 billion tons in 2019, with an average annual growth rate of 2.71%. Among them, Qinghai was the first to reach a carbon sink surplus, and Shandong was the largest in net carbon emissions. After incorporating economic factors into the model and revising the carbon offset benchmark value, China’s net carbon emissions dropped to a greater extent. In terms of geographic distribution, large agricultural provinces (Henan, Jiangxi, etc.) or economically underdeveloped regions (Gansu, Sichuan, etc.) should receive carbon ecological compensation, and energy-consuming provinces (Shaanxi, Shanxi, Inner Mongolia, etc.) should pay corresponding carbon ecological compensation costs.

(4) From the perspective of carbon compensation value, there is a large spatial difference in the value of carbon compensation between provinces in China. By incorporating the economic contribution coefficient and the price of carbon emission rights into the model, it is found that provinces such as Guangdong, Beijing, and Jiangsu should obtain corresponding carbon ecological compensation. However, Shandong, Inner Mongolia, Liaoning, Shanxi and other provinces should pay corresponding carbon compensation fees because their economic structure is dominated by heavy industries, which cause greater pollution to the environment.

(5) Regional carbon ecological compensation can be studied from different spatial scales, such as countries, provinces, cities, and villages. The size of the research object selection range determines the different research structures. Generally speaking, the smaller the research range, the more accurate the result will be. This paper uses the land carbon balance method to study the carbon emissions of China’s 30 provinces. The purpose is to measure the carbon emissions of each province from a macro level, so as to provide specific results for the transformation of the economic structure and industrial development of each province. In the future, the author will further refine the research objects, carry out horizontal carbon compensation studies from a smaller spatial scale, and combine certain field investigations to ensure that carbon compensation is more feasible and operable.

### Policy Recommendations

(1) China should give full play to the role of the government and the market to promote the establishment of a carbon ecological compensation system with Chinese characteristics. First, China should establish a regional horizontal carbon compensation mechanism led by the government, actively broaden compensation channels, promote the formation of a compensation system guided by the government and market participation, and give full play to the decisive role of the market in resource allocation. Second, China should build a fiscal and taxation policy system for the sustainable development of ecological compensation, formulate and improve laws and regulations on ecological compensation, and scientifically formulate a standard system related to ecological compensation, including value evaluation, compensation plans, and implementation effects. At the same time, the government should also strengthen its energy strategy and ecological environment constraints, take response to global climate change and achieve low-carbon development as important goals and restrict red lines, and implement a low-carbon-oriented differentiated regional assessment mechanism for local governments.

(2) China should continue to optimize and improve the allocation and transaction system of ecological property rights, and improve the efficiency of ecological compensation. It is necessary to further carry out a comprehensive survey of natural resource assets, clarify the ownership of natural resource assets, and determine the stakeholders of the ecological compensation mechanism. Second, the government should continue to improve the arable land compensation system, establish a green ecology-oriented subsidy system for agricultural ecological governance, and actively promote the paid use and trading system of carbon emission rights, the ecological compensation mechanism for forestry carbon sinks, and the carbon emission rights mortgage financing system. Third, China should improve the ecological environment rights and interests market trading mechanism, establish a national carbon trading market, and accelerate the establishment of a natural resource property right system with clear ownership, clear rights and responsibilities, smooth circulation, strict protection, and effective supervision.

(3) China should improve the compensation mechanism for ecological product protection. The ecological protection compensation mechanism is essentially a reasonable compensation for the opportunity cost of the ecological product supply area to fulfill the responsibility of ecological protection. First, the government should further clarify the objects, standards, and models of compensation for ecological protection and environmental damage in accordance with the principles of “who pollutes, who pays” and “who protects, who benefits”. The second is that the government must accelerate the improvement of the vertical ecological protection compensation system, improve the transfer payment fund distribution mechanism for key ecological function areas, but also accelerate the establishment of a horizontal ecological protection mechanism between provinces, and improve the benefit distribution and risk sharing mechanism. Third, the government should actively explore diversified ecological compensation methods, actively learn from carbon trading, carbon market and other typical cases, broaden the channels for transforming ecological resources into ecological capital, and gradually form a pattern of multi-participation and market-oriented operation.

(4) All provinces should adhere to the optimization of land development and utilization, establish the main functional areas based on carbon balance accounting, and restrict the development function. The government must always prioritize the protection of arable land, strengthen the management and control of land-use planning, strictly control the occupation of arable land for construction land, and promote the economical and intensive use of land. At the same time, the government should give full play to the complementary advantages of capital resources in economically developed regions and resource-rich regions, and establish a revenue adjustment and distribution mechanism. The government should also continue to improve the ecological and environmental health risk management and control environmental policy system, and decompose the responsibility for ecological and environmental protection to administrative regions at all levels, so as to fundamentally ensure the sound development of the ecological environment.

## Figures and Tables

**Table 1:** China’s major economic crop coefficient, water content and carbon absorption rate.

Crop Type	Economic Coefficient	Moisture Content	Carbon Absorption Rate
Wheat	0.28~0.46	0.12	0.49
Corn	0.45~0.53	0.13~0.14	0.47
Cereal	0.35~0.45	0.13~0.15	0.41
Beans	0.20~0.30	0.12~0.13	0.45
Tubers	0.60~0.75	0.13	0.42
Cotton	0.30~0.40	0.08	0.45
Tobacco Leaf	0.50	0.08	0.45
Oil Crops	0.33~0.45	0.08~0.10	0.45
Hemp	0.33~0.45	0.13	0.45
Sugar Crops	0.33~0.45	0.13	0.41

**Table 2:** Total carbon absorption of agricultural products by provinces in China (Mt).

Province	2010	2015	2019	Province	2010	2015	2019
Beijing	48.21	25.87	11.77	Henan	2503.35	2778.36	3044.23
Tianjin	68.42	75.47	90.12	Hubei	1043.22	1198.17	1188.64
Hebei	1308.58	1472.91	1598.15	Hunan	1173.62	1243.24	1218.98
Shanxi	450.44	503.92	523.48	Guangdong	983.83	1064.42	1015.37
Inner Mongolia	937.10	1265.55	1710.46	Guangxi	3066.69	3259.04	3185.90
Liaoning	718.35	798.13	998.60	Hainan	205.97	164.99	96.75
Jilin	1135.30	1453.14	1564.45	Chongqing	462.38	465.27	436.47
Heilongjiang	2022.37	2482.32	2948.48	Sichuan	1382.21	1458.17	1512.48
Shanghai	44.99	41.04	35.47	Guizhou	478.30	551.48	467.84
Jiangsu	1301.53	1420.03	1477.46	Yunnan	1271.42	1471.02	1343.92
Zhejiang	330.16	317.65	255.63	Shaanxi	496.09	523.25	517.84
Anhui	1328.91	1515.38	1682.56	Gansu	387.94	491.60	489.58
Fujian	289.69	290.83	219.66	Qinghai	51.25	49.71	48.30
Jiangxi	783.69	864.10	862.28	Ningxia	148.94	153.19	147.71
Shandong	1966.06	2110.33	2348.05	Xinjiang	779.91	953.58	1018.07

**Table 3:** Total carbon emissions from cultivated land by provinces in China (Mt).

Province	2010	2015	2019	Province	2010	2015	2019
Beijing	20.56	17.94	16.60	Henan	234.78	240.68	233.45
Tianjin	24.25	23.21	22.33	Hubei	158.68	163.13	153.07
Hebei	191.56	188.47	174.95	Hunan	158.39	171.20	163.66
Shanxi	65.93	73.42	71.45	Guangdong	121.68	124.12	110.45
Inner Mongolia	117.15	124.12	126.48	Guangxi	95.02	103.28	102.21
Liaoning	94.77	89.98	88.43	Hainan	42.78	40.55	31.92
Jilin	88.52	100.79	98.05	Chongqing	46.69	45.82	44.91
Heilongjiang	172.79	221.94	234.23	Sichuan	127.34	131.14	130.81
Shanghai	21.88	20.15	19.30	Guizhou	57.28	57.15	57.17
Jiangsu	171.93	169.92	170.94	Yunnan	88.58	100.62	98.98
Zhejiang	87.72	82.65	72.55	Shaanxi	60.01	59.36	59.89
Anhui	179.63	200.38	193.73	Gansu	75.45	93.62	75.24
Fujian	70.86	72.32	66.41	Qinghai	21.10	19.62	19.76
Jiangxi	123.99	122.37	107.14	Ningxia	28.23	29.40	29.83
Shandong	245.87	239.89	232.27	Xinjiang	128.75	166.95	166.65

**Table 4:** Total carbon emissions from construction land by province in China (Bt).

Province	2010	2015	2019	Province	2010	2015	2019
Beijing	12.92	11.14	10.15	Henan	61.44	62.68	51.92
Tianjin	18.44	19.45	19.60	Hubei	36.22	35.55	37.46
Hebei	83.92	88.26	93.31	Hunan	28.60	31.16	31.63
Shanxi	70.12	83.58	113.26	Guangdong	57.56	61.64	64.77
Inner Mongolia	60.66	78.36	104.60	Guangxi	17.39	22.06	30.25
Liaoning	67.94	68.63	81.42	Hainan	5.24	7.16	7.78
Jilin	25.33	24.96	26.07	Chongqing	15.63	16.35	13.99
Heilongjiang	34.16	36.23	35.33	Sichuan	33.82	34.54	30.25
Shanghai	26.76	26.40	26.34	Guizhou	23.28	27.73	29.23
Jiangsu	68.26	83.16	83.59	Yunnan	23.93	20.42	24.97
Zhejiang	42.96	43.77	46.74	Shaanxi	34.13	47.35	53.47
Anhui	31.45	39.13	42.67	Gansu	17.30	20.37	21.29
Fujian	22.70	27.25	32.13	Qinghai	3.98	5.17	6.06
Jiangxi	17.57	21.60	24.37	Ningxia	13.29	20.63	30.68
Shandong	109.21	132.66	148.86	Xinjiang	27.82	47.92	59.24

**Table 5:** Carbon compensation benchmark values of China’s provinces (1000 Mt).

Province	2010	2015	2019	Province	2010	2015	2019
Beijing	−125.55	−164.03	−225.60	Henan	−26.60	−29.93	−54.70
Tianjin	−43.51	−52.70	−31.44	Hubei	−44.17	−59.73	−72.33
Hebei	88.67	95.86	105.46	Hunan	−59.35	−67.65	−75.06
Shanxi	82.62	104.75	149.62	Guangdong	−79.90	−80.68	−93.46
Inner Mongolia	257.34	353.60	497.81	Guangxi	−55.29	−48.46	−25.81
Liaoning	119.03	118.59	160.59	Hainan	−42.16	−36.54	−39.07
Jilin	−8.82	−15.04	2.13	Chongqing	−57.08	−74.99	−108.19
Heilongjiang	15.75	20.45	26.68	Sichuan	−62.14	−72.63	−104.16
Shanghai	−66.81	−68.59	−86.73	Guizhou	−11.08	−15.73	−21.78
Jiangsu	−45.66	−37.16	−47.15	Yunnan	−34.61	−54.32	−59.68
Zhejiang	−62.41	−65.62	−73.03	Shaanxi	−0.54	17.59	23.22
Anhui	−32.19	−28.04	−38.12	Gansu	−15.14	−10.55	−9.45
Fujian	−68.69	−68.12	−76.03	Qinghai	−51.33	−48.11	−41.64
Jiangxi	−56.73	−54.56	−57.52	Ningxia	69.12	119.54	186.92
Shandong	93.93	127.03	154.09	Xinjiang	31.40	95.17	128.46

**Table 6:** Calculation of spatial carbon offset value in China’s provinces (100 million yuan).

Province	2010	2015	2019	Province	2010	2015	2019
Beijing	−16.04	−21.48	−10.93	Henan	−5.56	−6.30	−4.06
Tianjin	−3.63	−4.96	−0.61	Hubei	−6.38	−10.04	−4.54
Hebei	16.37	16.26	5.07	Hunan	−8.61	−11.12	−4.09
Shanxi	6.88	7.61	3.49	Guangdong	−33.27	−33.43	−13.78
Inner Mongolia	27.18	35.87	11.73	Guangxi	−4.79	−4.63	−0.75
Liaoning	19.88	19.34	5.48	Hainan	−0.79	−0.77	−0.28
Jilin	−0.69	−1.20	0.03	Chongqing	−4.09	−6.71	−3.50
Heilongjiang	1.48	1.75	0.50	Sichuan	−9.66	−12.42	−6.65
Shanghai	−10.38	−9.81	−4.53	Guizhou	−0.46	−0.94	−0.50
Jiangsu	−17.12	−14.83	−6.43	Yunnan	−2.26	−4.21	−1.90
Zhejiang	−15.66	−16.01	−6.24	Shaanxi	−0.05	1.80	0.82
Anhui	−3.60	−3.51	−1.94	Gansu	−0.56	−0.41	−0.11
Fujian	−9.16	−10.07	−4.41	Qinghai	−0.63	−0.66	−0.17
Jiangxi	−4.85	−5.19	−1.95	Ningxia	1.06	1.98	0.96
Shandong	33.30	45.53	15.00	Xinjiang	1.55	5.05	2.39

## Data Availability

Not applicable.
